# Fucoxanthin, a Marine-Derived Carotenoid from Brown Seaweeds and Microalgae: A Promising Bioactive Compound for Cancer Therapy

**DOI:** 10.3390/ijms21239273

**Published:** 2020-12-04

**Authors:** Sarah Méresse, Mostefa Fodil, Fabrice Fleury, Benoît Chénais

**Affiliations:** 1 EA 2160 Mer Molécules Santé, Le Mans Université, F-72085 Le Mans, France; Sarah.Meresse@cnrs-orleans.fr (S.M.); Mostefa.Fodil@univ-lemans.fr (M.F.); 2 UMR 6286 CNRS Unité Fonctionnalité et Ingénierie des Protéines, Université de Nantes, F-44000 Nantes, France; Fabrice.Fleury@univ-nantes.fr; 3 UMR 7355 CNRS Immunologie et Neurogénétique Expérimentales et Moléculaires, F-45071 Orléans, France

**Keywords:** angiogenesis, apoptosis, cancer, cell growth arrest, DNA repair, EMT, fucoxanthin, inflammation, invasion, migration

## Abstract

Fucoxanthin is a well-known carotenoid of the xanthophyll family, mainly produced by marine organisms such as the macroalgae of the fucus genus or microalgae such as *Phaeodactylum tricornutum*. Fucoxanthin has antioxidant and anti-inflammatory properties but also several anticancer effects. Fucoxanthin induces cell growth arrest, apoptosis, and/or autophagy in several cancer cell lines as well as in animal models of cancer. Fucoxanthin treatment leads to the inhibition of metastasis-related migration, invasion, epithelial–mesenchymal transition, and angiogenesis. Fucoxanthin also affects the DNA repair pathways, which could be involved in the resistance phenotype of tumor cells. Moreover, combined treatments of fucoxanthin, or its metabolite fucoxanthinol, with usual anticancer treatments can support conventional therapeutic strategies by reducing drug resistance. This review focuses on the current knowledge of fucoxanthin with its potential anticancer properties, showing that fucoxanthin could be a promising compound for cancer therapy by acting on most of the classical hallmarks of tumor cells.

## 1. Introduction

Carotenoids are colored and natural pigments widely distributed in nature. They include more than 1100 molecules [1], divided into two classes: xanthophylls, which contain oxygen, and carotenes, which are pure hydrocarbons. In photosynthetic organisms such as plants and algae, carotenoids have two major roles: they absorb energy for photosynthesis and also protect chlorophyll from photodamage [2]. At a structural level, xanthophyll pigments are close to each other, they share a long carbon chain containing one or more oxygen atoms, which differentiates them from carotenes. These carotenoids pigments are well-known for their antioxidant and anti-inflammatory properties [3,4,5], but they also display some potential anticancer effects.

**Figure 1:**
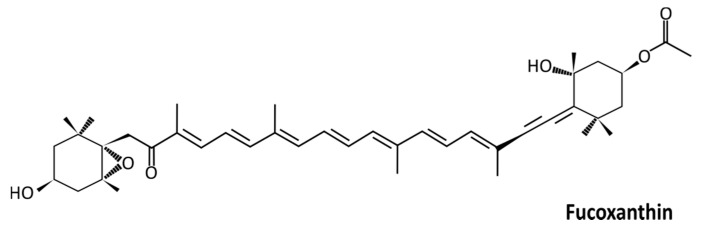
Structure of fucoxanthin (3′-acetoxy-5,6-epoxy-3,5′-dihydroxy-6′,7′-didehyro-5,6,7,8,5′,6′-hexahydro-β,β-carotene-8-one; C_42_H_58_O_6_; 658.91 g/mol).

First isolated in 1914 by Willstätter and Page, fucoxanthin (Figure 1) is an orange-colored xanthophyll pigment derived from brown algae and microalgae [6,7,8]. It is found in high content in taxons such as Phaeophyceae, Haptophyta, Bacillariophyceae, and Chrysophyceae, and to a lesser extent in Rhodophyta, Raphidophyceae, and Dinophyta [9]. Fucoxanthin is one of the most abundant carotenoid pigments, which contributes to more than 10% of the estimated total carotenoid production in nature, particularly in the marine environment [10].

Popular sources of fucoxanthin include macroalgae such as *Laminaria japonica*, *Eisenia bicyclis*, and the well-known brown seaweed Wakame (*Undaria pinnatifida*), as well as diatoms microalgae such as *Phaeodactylum tricornutum* [11] (Table 1). Fucoxanthin exhibit several biological activities that are beneficial to human health including antioxidant, anti-inflammatory, anti-obesity, anti-diabetic, anti-angiogenic, and anticancer properties [12,13,14,15,16,17].

## 2. Absorption and Metabolites of Fucoxanthin

**Figure 2:**
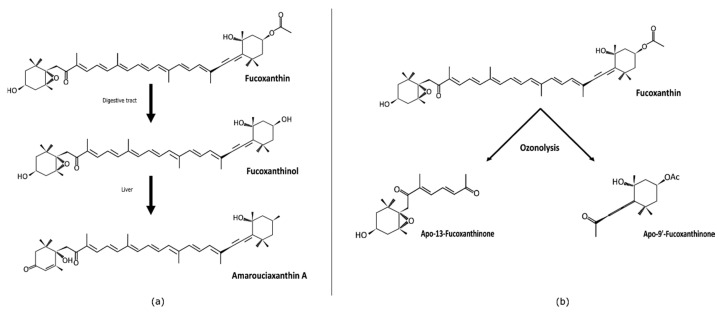
Natural (**a**) and synthetic (**b**) metabolites of fucoxanthin (adapted from Komba et al., 2018 [47]).

Being hydrophobic, carotenoids are absorbed in the intestine following the same path as dietary fats. Fucoxanthin has an allenic bond, a polyene chain, an acetyl, and a β,γ-epoxy ketone group (Figure 1). There is no symmetry between the two six-membered ring derivatives bound by the polyene chain: one has an allenic bond, the second has a β,γ-epoxy ketone group. In vivo, the bioactive forms of fucoxanthin are fucoxanthinol and/or amarouciaxanthin. When ingested, fucoxanthin is mainly metabolized to fucoxanthinol in the gastrointestinal tract by digestive enzymes such as lipase and cholesterol esterase by hydrolysis [45], and it is further converted to amarouciaxanthin A in the liver [46] (Figure 2a). Research on the possible effects of amarouciaxanthin A remains insufficient in the context of cancer. Nonetheless, beneficial health effects of fucoxanthin and fucoxanthinol are well described.

Two other synthetic metabolites could be obtained by chemical degradation (ozonolysis) of fucoxanthin apo-9′-fucoxanthinone [48] and apo-13-fucoxanthinone [49] (Figure 2b). Ozonolysis cleaved the polyene chain of fucoxanthin into two types of cyclohexyl derivatives, the first one with a β,γ-epoxy ketone group and the second one with an allenic bond. Caco-2 cells (human colorectal carcinoma) were treated with compounds resulting from fucoxanthin degradation to determine the correlation between structure and activity. From this study, the complete structure of fucoxanthin appears not indispensable for its antiproliferative effect, which could be achieved by a partial structure of the molecule [29,47].

Given the high safety of fucoxanthin and its metabolites, and their significant bioactivities and pharmacological effects, fucoxanthin has the potential to become a promising nutritional ingredient and a potential medicinal constituent for human health [50]. Here, we will discuss fucoxanthin as a potential treatment and/or preventive agent of cancer development and aggressiveness.

## 3. Antiproliferative Effects of Fucoxanthin through Cell Cycle Arrest in Cancer Cells

The antiproliferative effect of fucoxanthin has been reported for several cancer cell lines (Table 2), including human leukemia cell lines, K562 and TK6 [51], adult T-cell leukemia [52], B-cell malignancies, including Burkitt’s lymphoma, Hodgkin’s lymphoma, and Epstein-Barr virus-immortalized B cells [19], primary effusion lymphoma (PEL), a rare type of non-Hodgkin’s lymphoma [53], human bladder cancer T24 cell line [54], melanoma cell lines (B16F10 cells) [55], human gastric adenocarcinoma MGC-803 cells [56], colon adenocarcinoma cell lines WiDr and HCT116 [57], LNCap and DU145 prostate cancer cells [58,59,60], human neuroblastoma GOTO cell line [61], human hepatocarcinoma HepG2 cell line [59,60,62,63], SK-Hep-1 human hepatoma cells [64], cervical cancer cells HeLa [65], and osteosarcoma cell lines [20]. The inhibition of cell proliferation by fucoxanthin is due to cell growth arrest at G0/G1 or G1 phase of the cell cycle [19,20,52,53,54,55,57,58,59,60,61,62,64,65].

In bladder and colon cancer cells, this arrest in the cell cycle is mediated by the upregulation of p21WAF1/Cip1, a cyclin-dependent kinase (CDK)-inhibitory protein [54,57]. The molecular mechanism of cell cycle arrest also involves the downregulation of cyclin D1 and CDK4 complex in B cell malignancies, bladder cancer, melanoma, and HepG2 hepatocarcinoma cell lines [19,54,55,62]. In bladder cancer cells, a decrease of CDK-2 and cyclin E was also observed [54], whereas a decrease of cyclin D2 appears in B cell malignancies and melanoma cells [19,55], and a decreased expression of CDK4, CDK6, and cyclin E could be found in adult T-cell Leukemia [52]. In the HepG2 cell line, Das et al. observed a downregulation of the kinase activity of cyclin D and CDK4 complex, responsible for the phosphorylation of Ser780 of retinoblastoma protein (pRb) [62]. Also, Kim et al. showed a clear lowering of the protein expression of phosphorylated pRb (phospho-pRb), cyclin D1, cyclin D2, and CDK4. They also observed an upregulation of the proteins p27Kip1 and p15INK4B in B16-F10 cells (melanoma) treated with fucoxanthin [55]. In contrast, in colon cancer cell lines, Das et al. have shown that G0/G1 phase arrest was mediated through the increased phosphorylation of pRB and upregulation of p21WAF1/Cip1 but not p27Kip1, and without changes in the protein levels of the D-types of cyclin and CDK4 [57]. In addition, the study of Satomi et al., using prostate cancer cell lines, suggested that GADD45A and Jun-N-terminal kinase (JNK) might be involved in fucoxanthin-induced G1 cell cycle arrest [58]. Curiously, only one study has reported a fucoxanthin-induced cell cycle arrest in G2/M phase, which was related to a decreased expression of cyclin B1 and survivin in human gastric adenocarcinoma MGC-803 cells [56].

The antiproliferative effect of fucoxanthin was confirmed in vivo by studies showing tumor growth arrest in the presence of fucoxanthin with several types of cancer (Table 3). For example, Mei et al. showed in vivo anti-lung cancer effect of fucoxanthin, and Kim et al. showed that the growth of tumor mass in B16F10 cells-implanted Balb/c mice has been significantly inhibited by the intraperitoneal administration of fucoxanthin [55,67].

**Figure 3:**
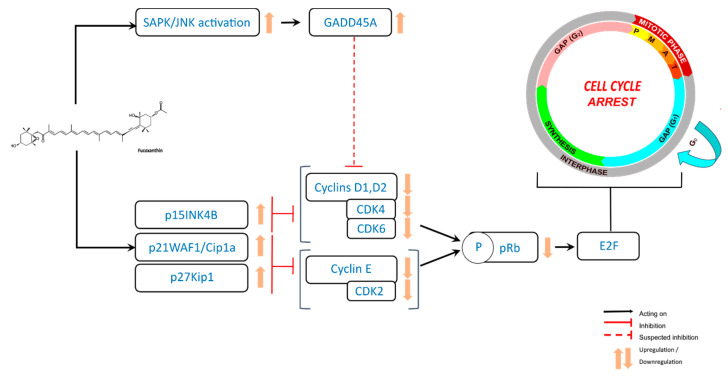
Antiproliferative effects of fucoxanthin through cell cycle arrest in cancer cells.

Finally, the molecular mechanism that supports the observed G0/G1 phase arrest appears dependent on the cancer cell type but mainly involves cyclin D1 and/or D2 and CDK4 downregulation (Figure 3). However, cyclin E and CDK2 or CDK6 could also be involved in some cell types.

## 4. Induction of Apoptosis and Autophagy in Cancer Cells

Autophagy and apoptosis are mechanisms involved in the development of a variety of cancers. Fucoxanthin has been highlighted to induce autophagy and apoptosis in cellular cell lines including HeLa, SGC-7901, and human cervical cancer [65,70,83] (Table 2). Anoikis is useful to maintain homeostasis in normal conditions. Anoikis is the name given to induction of apoptosis in cells upon loss of attachment to the extracellular matrix and cellular neighborhood [95]. Cancer cells are resistant to anoikis, favoring metastasis and new tumor growth [95,96]. In order to include fucoxanthin in a potential therapeutic strategy, some pathways have been studied and identified to be involved in those cytotoxic effects.

### 4.1. Apoptosis

Fucoxanthin inhibited the growth of U251 human glioma cells in a time- and dose-dependent manner by induction of apoptosis with poly-ADP-ribose polymerase (PARP) cleavage and caspase activation [84]. Furthermore, fucoxanthin treatment of glioma cells caused disturbance of mitogen-activated protein kinases (MAPKs) and phosphoinositide-3 kinase (PI3K)-Akt pathways, in a time-dependent manner. This was highlighted by the activation by phosphorylation of Thr183-JNK, Thr180-p38, and Thr202 of extracellular signal-regulated kinase (ERK) and, in contrast, the inactivation of Akt by phosphorylation of Ser473 [84]. However, the inhibition of reactive oxygen species (ROS) by the antioxidant glutathione counteracts fucoxanthin effects, including the alteration of MAPKs and PI3K-Akt signaling, DNA damage, and cytotoxicity and cell apoptosis. This indicates that ROS production acts as an early apoptotic event and is involved in the fucoxanthin-triggered anticancer mechanism [84].

Results consistent with the data described above were obtained using the human U87 glioma cell line [85]. In the human hepatocarcinoma HepG2 cell line, apoptosis induction by fucoxanthin was correlated to a decreased expression of genes coding antioxidant enzymes superoxide dismutase (*SOD1*, *SOD2*) and catalase (*CAT*) and downregulation of signaling pathways (*AKT*, *JNK*, *ERK1/2*) [69]. The involvement of ROS in fucoxanthin-induced apoptosis was also reported for HL-60 leukemia and fucoxanthin-induced cytotoxicity, and apoptosis was suppressed by the ROS scavenger N-acetylcysteine (NAC) [25]. In HL-60 cells, fucoxanthin induced the cleavage of caspase-3, caspase-7, and PARP, and a decrease of the apoptosis inhibitor Bcl-xL. Moreover, the pre-treatment of cells with NAC prevented these effects of fucoxanthin [25]. In accordance, fucoxanthin increased the apoptosis in human cervical cancer cells, namely HeLa, SiHa, and CaSki cell lines, by targeting the PI3K/Akt/nuclear factor-kappa B (NF-κB) signaling pathway [83]. Fucoxanthin was also shown to synergize with tumor necrosis factor-related apoptosis-inducing ligand (TRAIL) to induce apoptosis in human cervical cancer cells [83].

Fucoxanthin also induces apoptosis in human EJ-1 and T24 bladder cancer cell lines [54,75]. In T24 cells, fucoxanthin-induced apoptosis was linked to a decrease in the expression level of mortalin, which is a stress regulator and a member of heat shock protein 70, followed by upregulation of cleaved caspase-3 at a high dose (40 µM) [54].

Fucoxanthin induced apoptosis of non-small cell lung cancer by modulating expression of p53, p21, Fas, p53 upregulated modulator of apoptosis (PUMA), Bcl-2, and caspase-3/8 [67]. In the B16F10 human melanoma cell line, fucoxanthin-induced apoptosis was associated with the downregulation of Bcl-xL, leading to the sequential activation of caspase-9, caspase-3, and PARP [55]. If most studies report the activation of caspase-3 as a hallmark of fucoxanthin-induced apoptosis, some discrepancies exist about the pro- and anti-apoptotic proteins. For example, fucoxanthin reduced the expression of Bax and Bcl-2 proteins, but not Bcl-xL, in the prostate cancer cell line PC-3 [77], while decreasing Bcl-xL in B16F10 melanoma cells [55]. In contrast, fucoxanthin has no effect on the protein level of Bcl-2, Bcl-xL, or Bax in HL-60 promyelocytic leukemia cells [97]. The decrease of survivin, an apoptosis inhibitory protein, expression was thought to be responsible for the induction of apoptosis by a high dose of fucoxanthin (75 µM) in human gastric adenocarcinoma MGC-803 cells [56].

Fucoxanthin (Table 2) and more importantly its metabolite fucoxanthinol (Table 4) are able to reduce NF-κB activation and induce apoptosis of the colorectal cancer cell line HCT116, breast cancer cell lines MCF-7 and MDA-MB-231, B-cell malignancies, and primary effusion lymphoma [19,53,78,80,98]. In all these studies, fucoxanthinol was reported to be more potent than its precursor fucoxanthin. This was also observed in the murine macrophages’ cell line RAW264.7, where high concentrations of both fucoxanthin and fucoxanthinol (>10 μM) induced apoptosis with activation of caspase 3/7 and suppression of the anti-apoptotic proteins, such as Bcl-xL and phospho-Akt.

**Figure 4:**
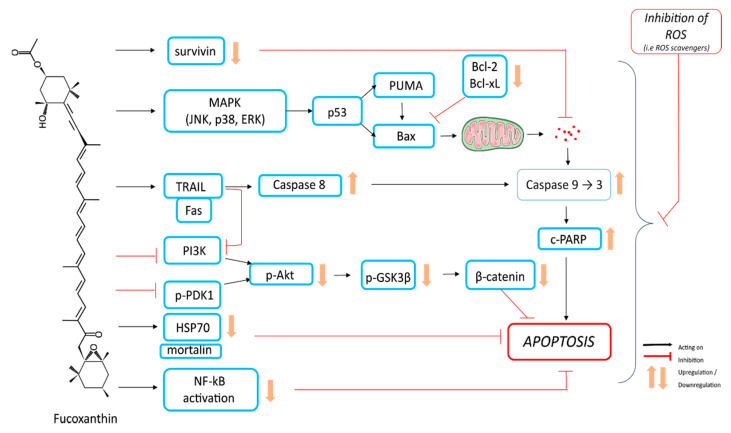
Induction of apoptosis in cancer cells by fucoxanthin.

Moreover, lower concentrations of fucoxanthin and fucoxanthinol (1–4 μM) have a cytoprotective effect, inducing nuclear factor (erythroid-derived 2)-like 2 (NFE2L2/Nrf2) transcription factor and heme oxygenase antioxidant enzyme expression, while a higher concentration activates NFE2L2/Nrf2 and induces apoptosis [100]. In human Saos-2 osteosarcoma cells, fucoxanthin and fucoxanthinol induce apoptosis, at least in part, by inhibiting Akt and activator protein-1 (AP-1) pathways [20]. Apoptosis of fucoxanthinol-treated Saos-2 cells was characterized by a reduced expression of survivin, X-linked inhibitor of apoptosis (XIAP), Bcl-2, and Bcl-xL, and associated with activation of caspases-3, 8, and 9 [20]. A similar pattern was observed in Adult T-cell Leukemia treated by fucoxanthin or fucoxanthinol, which was twice as potent [52]. In addition, fucoxanthinol inhibited the phosphorylation of Akt, phosphoinositide-dependent kinase 1, and the downstream glycogen synthase kinase 3β, leading to the downregulation of β-catenin [20]. In HL-60 leukemia cells, fucoxanthinol also demonstrated a greater pro-apoptotic effect than fucoxanthin, characterized by a decreased level of Bcl-2 protein [74]. Similarly, a decrease of Bcl-2 protein together with increased DNA fragmentation was observed in CaCo-2 colon cancer cells, which was partially inhibited by a caspase inhibitor Z-VAD-fmk [72]. Effects of fucoxanthin on apoptosis are summarized in Figure 4.

The in vitro effects of fucoxanthin on cell death are clearly determined and indicate a cytotoxicity, even at low doses, in cancer cell lines. Those effects have also been observed in vivo, in mice bearing xenografted sarcoma 180 (S180). At 50 and 100 mg/kg, fucoxanthin significantly inhibited the growth of sarcoma and induced apoptosis, as demonstrated by the decrease of Bcl-2 expression and a clear increase of cleaved caspase-3 [94]. Moreover, the same authors also reported a clear lowering of survivin, vascular endothelial growth factor (VEGF), epidermal growth factor receptor (EGFR), signal transducer and activator of transcription 3 (STAT3) protein expression, and phosphorylated STAT3 level [94].

### 4.2. Anoikis

**Figure 5:**
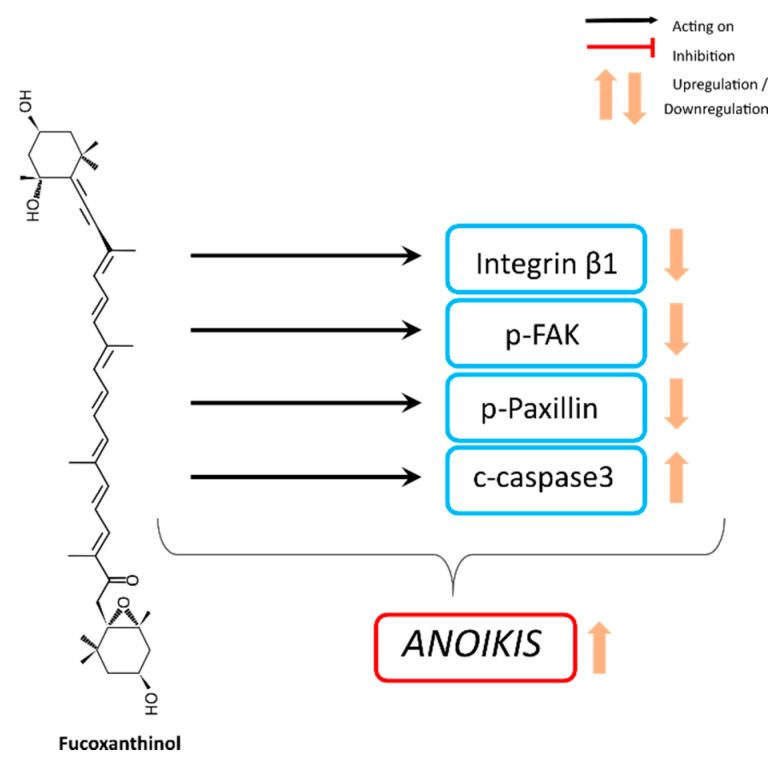
Anoikis induction by fucoxanthinol in cancer cells.

A low concentration (2.5 µM) of the fucoxanthin metabolite fucoxanthinol induced anoikis, i.e., anchorage-dependent apoptosis, through integrin β1 signal suppression in human DLD-1 colorectal cancer cells [99] (Table 4). The anoikis characteristics were further described in DLD-1 cells as low or null level of integrin β1, low or null level of phosphorylated focal adhesion kinase (p-FAK–Tyr397), or low or null level of phosphorylated Paxillin (Tyr31), and with a high level of cleaved caspase-3 [92]. In vitro effects (Figure 5) have been confirmed in vivo using an azoxymethane/dextran sodium sulfate carcinogenic mouse model, showing that fucoxanthin ingestion (30 mg/kg body weight) significantly diminished the number and size of polyps compared with untreated control mice and that cells with anoikis features were in colonic crypts significantly increased in fucoxanthin-treated mice [92] (Table 3).

### 4.3. Autophagy

**Figure 6:**
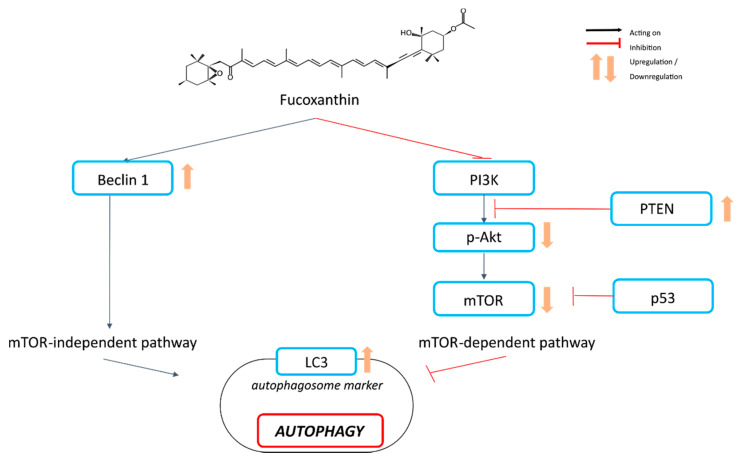
Induction of autophagy in cancer cells by fucoxanthin.

Treatment of HeLa cells with fucoxanthin (10–80 μM) increased the protein expression of microtubule-associated protein 1A/1B-light-chain 3 (LC3), an autophagosome marker, and Beclin-1, the initiation factor for autophagosome formation [65]. Furthermore, fucoxanthin decreased the levels of phospho-Akt and its downstream proteins p53, p70S6K, and mechanistic target of rapamycin (mTOR) in a dose-dependent manner, and increased the expression of the so-called “phosphatase and tensin homolog” PTEN protein. This suggests that the cytotoxic effect of fucoxanthin is dependent on autophagy in HeLa cells via inhibition of the Akt/mTOR signaling pathway [65]. In gastric cancer SGC7901 cells, fucoxanthin (12.5–50 µM) significantly inhibits the viability of SGC-7901 cells by inducing both autophagy and apoptosis just as effectively, as shown by the downregulation of Bcl-2 and upregulation of Beclin-1, LC3, and cleaved caspase-3. In addition, it appears that fucoxanthin-induced autophagy (Figure 6) occurs prior to apoptosis and may be a promoter of apoptosis [70]. Those two processes are related with each other. Indeed, an increased level of caspase-3 plays a key role in apoptosis.

## 5. Involvement in DNA Damages

Cells are continuously exposed to exogenous and endogenous sources of ROS. The antioxidant properties of fucoxantin limit ROS-induced DNA damage. Thus, in human fibroblast, Heo and Jeon have shown that high concentrations of fucoxanthin (i.e., 5–250 µM) extracted from the marine algae *Sargassum siliquastrum* decreased the UV B-induced DNA-damage [33]. In cancer cells, fucoxanthin (at 6 µM) displays a protective effect against UV-induced DNA damage, as well as heavy metal and heat-induced protein misfolding and aggregation of proteins, in rat glioma cells [82] (Table 2). In addition, Fucoxanthin from *Sargassum* sp. (20 µM) also shows some protective effects toward bleomycin-induced DNA damage in MCF-7 breast cancer cells [79].

However, under other experimental conditions, fucoxanthin and its metabolites can lead to inducing DNA damages, which can be used for anticancer therapy. Konishi et al. report an augmentation of DNA damage in HL-60 cells from 12.5 and 6.25 µM when cells were treated respectively with fucoxanthin and fucoxanthinol [74] (Table 4). A similar effect was also reported for MCF-7 and Caco-2 cells, and 12.5 µM fucoxanthinol significantly enhances DNA damages, while the same effect was obtained with 25 µM fucoxanthin [74]. These data confirm that once metabolized by the liver, this biomarine compound becomes more active and beneficial for human health within cancer cells. Interestingly, in colon cancer cell lines HCT116 and HT29, fucoxanthin reduced cell viability and induced DNA damage, but without an apparent effect in normal colon cell line CCD-18Co, except at high concentrations (50 and 100 µM). This study also reported that fucoxanthin enhanced the cytotoxic effect of 5-fluoro-uracile in colon cancer cells [73].

In human U51 glioma cells, fucoxanthin was shown to time-dependently induce ROS-mediated DNA damage, as evidenced by the activation of “ataxia-telangiectasia mutated” protein (ATM), “ataxia telangiectasia and Rad3-related” protein (ATR), and p53 by phosphorylation of Ser1981-ATM, Ser428-ATR, and Ser15-p53 respectively, as well as by the phosphorylation of Ser139 of histone H2AX [84]. Moreover, in the human hepatocarcinoma cell line HepG2, fucoxanthin pretreatment (10 µM, 24 h) significantly attenuated cisplatin-induced mRNA expression of DNA repair genes, namely excision repair cross complementation 1 (ERCC1), which is involved in the pathway of DNA nucleotide excision repair and thymidine phosphorylase, a central protein in the pyrimidine nucleoside salvage pathway. Fucoxanthin-modulated mRNA expression leads to improvement of chemotherapeutic efficacy of cisplatin [68]. It is noteworthy that the above-mentioned downregulation of survivin [94] may be linked to the impairment of DNA repair pathways [101,102].

Thus, fucoxanthin may have either a protective or promoting effect with respect to DNA damage depending on the cell types and perhaps the dose used. Further investigations are necessary to identify any impact of fucoxanthin on DNA repair mechanisms.

## 6. Inhibition of Metastasis-Related Migration, Invasion and Epithelial–Mesenchymal Transition

Metastasis is one of the major fundamental mechanisms of invading cancers [103]. The most destructive forms of cancers infiltrate secondary tissues and can escape the immune response. This allows to replace the tumor microenvironment for metastatic colonization by adapting to supportive niches and surviving as latent tumor-initiating cells [104]. Besides, epithelial–mesenchymal transition (EMT) is one of the most important processes controlling metastasis and is hardly regulated by activation of Wnt/β-catenin signaling [105]. Many proteins contribute to EMT, such as the mesenchymal structural regulator vimentin, the adhesion/migration marker fibronectin, and the extracellular matrix degrading proteins, i.e., matrix metalloproteinases (MMPs) [106,107].

**Figure 7:**
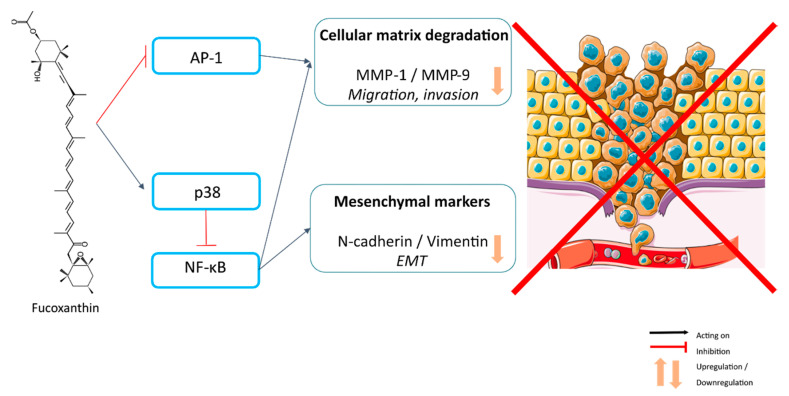
Inhibition of metastasis-related migration, invasion, and epithelial–mesenchymal transition by fucoxanthin.

Fucoxanthin was found to possess strong anticancer and anti-metastatic activities that work irrespective of the p53 status of cancer cells and cause a decrease in hallmark proteins associated with the metastatic spread of cancer cells at doses that were relatively safe to the normal cells [71] (Table 2). The development of osteosarcoma in mice inoculated with osteosarcoma cells was inhibited by a treatment with fucoxanthin [20] (Table 3). Fucoxanthinol, a metabolite of fucoxanthin, inhibited the cell migration and invasion of osteosarcoma cells, and reduced MMP-1 expression and AP-1 signaling [20] (Figure 7). In addition, fucoxanthinol significantly suppressed sphere-forming activity, migration, and invasion of human colorectal cancer cell lines HT-29 and HCT116 in a dose-dependent manner [99] (Table 4). Moreover, the expression of N-cadherin and vimentin was suppressed by a treatment with 50 μM of fucoxanthinol as well as the activation of integrin signaling linked to EMT inhibition [99]. Fucoxanthin also inhibited the expression and secretion of MMP-9, which plays a critical role in tumor invasion and migration and suppressed invasion of highly metastatic B16-F10 melanoma cells [30], as well as the human glioblastoma cell line U87 [85]. This effect, in glioblastoma cell line U87, is dependent on the p38 MAPK signaling [85]. Furthermore, fucoxanthin decreased the expression of the cell surface glycoprotein CD44 and C-X-C motif chemokine receptor-4 (CXCR4), which are involved in migration, invasion, and adhesion of cancer cells to endothelial cells. Indeed, the adhesion of B16-F10 melanoma cells to the endothelial cells was significantly inhibited by fucoxanthin [30]. Moreover, in vivo metastasis was reduced by fucoxanthin, as shown by a significant reduction of tumor nodules in an experimental lung metastasis in vivo assay [30] and glioblastoma xenografts [85].

Taken together, these results show the potential of fucoxanthin in reducing invasion, EMT, and finally, metastasis in some cancer models.

## 7. Anti-Angiogenic Effect of Fucoxanthin

Angiogenesis could be defined as the process of remodeling the primitive network of blood vessels and its growth into a complex network that is regulated by the balance between pro- and anti-angiogenic molecules. During this process, vascular endothelial cells secrete proteases and then migrate through the extracellular matrix, proliferate, and differentiate into new blood vessels [108]. Pathological angiogenesis is involved in many diseases, including rheumatoid arthritis, atherosclerosis, diabetic retinopathy, and cancer [109]. Angiogenesis is mandatory for tumor progression because newly formed blood vessels are needed to supply oxygen and nutrients, which are essential to the growing tumor, and to remove waste products. During angiogenesis, cancer cells secrete various pro-angiogenic factors such as VEGF, platelet-derived growth factor, and fibroblast growth factor 2 (FGF-2) [110]. Also, the metastasis process depends on angiogenesis, as tumor cells migrate from the primary tumor and grow in distant target organs. Few studies highlighted the anti-angiogenic potential of fucoxanthin [14,87,88] (Table 2 and Table 3). First, fucoxanthin was shown to inhibit human umbilical vein endothelial cells’ (HUVEC) proliferation and tube formation, but without a significant effect on HUVEC chemotaxis [87]. Fucoxanthin (10–20 µM) also suppressed the development of blood vessel-like structures from CD31-positive cells, and then may suppress the differentiation of endothelial progenitor cells into endothelial cells involving new blood vessel formation. In addition, fucoxanthin and its derivative fucoxanthinol suppressed, in a dose-dependent manner, micro-vessel outgrowth in an ex vivo angiogenesis assay using a rat aortic ring [87] (Table 4). The molecular mechanism of this anti-angiogenic effect of fucoxanthin involves the downregulation of the mRNA level of FGF-2 and its receptor (FGFR-1) as well as their trans-activation factor, EGR-1, as shown in HUVEC treated by fucoxanthin [14]. Moreover, fucoxanthin downregulates the FGF-2-mediated phosphorylation of signaling proteins such as ERK1/2 and Akt, which leads to the repression of endothelial cells’ migration as well as their differentiation into tube-like structures on Matrigel^®^ [14]. Another marine-derived compound, siphonaxanthin, structurally close to fucoxanthin, showed similar effects, causing a dose-dependent inhibition on HUVECs proliferation and tube formation (at concentration of 2.5, 10, and 25 μM). Furthermore, an ex vivo study also indicated the decrease of micro-vessel outgrowth from rat aortic fragments [14].

More recently, Wang et al. used fucoxanthin (25–100 µM) extracted from *Undaria pinnatifida* with human lymphatic endothelial cells (HLEC) and showed inhibition of proliferation, migration, and formation of tube-like structures [88]. In human breast cancer MDA-MB-231 cells, fucoxanthin also suppressed the malignant phenotype and decreased tumor-induced lymphangiogenesis when used in combination with a conditional medium culture system [88]. In vivo, using a MDA-MB-231 nude mouse model of breast cancer, high doses of fucoxanthin (100–500 µM) decreased micro-lymphatic vascular density, suggesting that fucoxanthin inhibits tumor-induced lymphangiogenesis in vitro and in vivo. At the cellular level, the mechanism of action of fucoxanthin involves decreased levels of VEGF-C, VEGF receptor-3, NF-κB, p-Akt, and p-PI3K in HLEC [88].

## 8. Anti-Inflammatory Effects of Fucoxanthin

Chronic inflammation has been associated with several stages of tumorigenesis, including cellular transformation, promotion, survival, proliferation, invasion, angiogenesis, and metastasis [111,112,113,114,115,116,117,118]. Cells responsible for cancer-associated inflammation are supposed to be genetically stable and poorly subjected to rapid emergence of drug resistance. Targeting inflammation may be an attractive strategy for cancer prevention and therapy [119].

Pro-inflammatory mediators including interleukins, tumor necrosis factor α (TNFα), prostaglandin E2, and nitric oxide contribute to the development of a variety of inflammatory diseases [120]. Natural products such as carotenoids have been used in the prevention of oxidative stress due to their antioxidant activities [120]. Despite the fact that the oxidative or anti-oxidative properties of fucoxanthin are discussed and seem to depend on the cellular context, this compound has been studied in an anti-inflammatory context in vivo and in vitro [121,122].

Ishikawa et al. showed, on adult T-cell leukemia—a fatal malignancy of T lymphocytes caused by human T-cell leukemia virus type 1 (HTLV-1), that fucoxanthin and fucoxanthinol inhibited cell viability and that the metabolite was twice as potent. Interestingly, uninfected cell lines and normal peripheral blood mononuclear cells were resistant to fucoxanthin and fucoxanthinol [52]. In contrast, other carotenoids, astaxanthin and β-carotene, had mild inhibitory effects on T-cell lines infected by HTLV-1. Otherwise, Lin et al. evaluated the anti-inflammatory effect of *D. salina* carotenoid extract, containing α-carotene and β-carotene on pseudo-rabies virus-infected RAW264.7 cells [123]. They found a downregulation of the expression of pro-inflammatory genes for interleukins (ILs) IL-1β, IL-6, and TNFα, but also monocyte chemoattractant protein-1 (MCP-1) in a concentration-dependent manner. This was correlated with a decrease of NF-κB activation by Toll-Like receptor-9, dependent on PI3K/Akt activation [123].

A recent study looked at the effects of the combination of fucoxanthin and rosmarinic acid (a bioactive compound from Lamiaceae plants) on inflammasome regulation. This combination improved anti-inflammatory effects from 5 µM each. Indeed, the inflammatory response is modulated through downregulation of inflammasome components such as “NOD-like receptor family, pyrin domain containing 3” (NLRP3), “apoptosis-associated speck-like protein” (ASC), caspase-1, and interleukins. These results suggest that fucoxanthin, in combination with rosmarinic acid, exerts anti-inflammatory effects by downregulating NRLP3-inflammasome and increasing the NFE2L2/Nrf2 signaling pathway in UVB-exposed HaCaT keratinocytes [15].

## 9. Fucoxanthin in Clinical Trials

Despite proven effects in animal studies, there are few clinical trials with pure algal metabolites, limited to those carried out with kahalalide F and fucoxanthin [124]. Most of the current clinical trials are aimed at confirming the effect of algae consumption, as extracts or fractions, on obesity and diabetes, but, to our knowledge, there are no clinical trials involving fucoxanthin in cancer treatment.

In 2008, Asai et al. detected fucoxanthinol at a concentration of 0.8 nM in plasma of individuals after the daily intake of the brown seaweed wakame (*Undaria pinnatifida*) containing 6.1 mg fucoxanthin for 1 week [125]. A 16-week clinical trial with 151 obese women using Xanthigen, a dietary supplement composed of brown seaweed extract containing 2.4 mg of fucoxanthin and pomegranate seed oil, was performed by Abidov et al. [126]. The results confirmed a significant weight loss and reduction of abdominal circumference in subjects whose body mass index (BMI) was over 30 kg/m^2^ [126].

Otherwise, Hashimoto et al. conducted a study on eighteen human volunteers who received an extract of kombu containing 31 mg of fucoxanthin, administered orally. Peripheral blood was collected before and after the treatment and plasma concentrations of fucoxanthinol were measured by high-pressure liquid chromatography, showing that a maximum concentration of 44.2 nM fucoxanthinol was obtained after 4 h. By contrast, the hepatic metabolite of fucoxanthinol, Amarouciaxanthin A, was not present in the volunteers’ plasma [127]. In the same context, Mikami et al. developed a simple and reproducible protocol for quantification of fucoxanthinol in human serum using liquid chromatography coupled with tandem mass spectrometry, which has a high sensitivity and a wide dynamic range. Using capsules of fucoxanthin, prepared by mixing edible brown seaweed (*Sargassum horneri*) oil and medium-chain triglyceride oil, it was confirmed that fucoxanthinol is a major metabolite of fucoxanthin present in human blood. In addition, although fucoxanthin supplementation does not affect visceral fat areas, it may decrease the level of glycated hemoglobin (HbA1c) in individuals carrying the thrifty allele of uncoupling protein-1 (UCP1-3826A/G) [128].

In 2013, Ren et al. investigated whether fucoxanthin or fucoidan (a polysulfated polysaccharide found in various species of brown seaweed), exhibited anti-thrombotic effects [129]. They prepared three types of capsules from the alga *Laminaria japonica*, containing 1 mg fucoxanthin, 400 mg fucoidan, and both, and administered it to 24 volunteers for 5 weeks. The dose of fucoidan or fucoidan + fucoxanthin significantly shortened the lysis time of the thrombus, as measured by a global thrombosis test in the blood, but fucoxanthin did not. Examining the mechanism, dietary fucoidan increased the production of H_2_O_2_ and the secretion of prostacyclin (PGI2), a potent inhibitor of platelet aggregation, while fucoidan was below the limit of detection in blood.

Recently, Hitoe and colleagues conducted a clinical trial of fucoxanthin supplementation in Japanese obese subjects [130]. They explored the effect of fucoxanthin (1 or 3 mg daily) in a double-blind placebo-controlled study. Capsules containing fucoxanthin or placebo capsules were administered for 4 weeks to male and female Japanese adults with a BMI of more than 25 kg/m^2^. In accordance with Reference [126], they observed a significant reduction of the relative (ratio versus before treatment) body weight, BMI, and visceral fat area in the 3 mg/day fucoxanthin group compared to the placebo group.

Other clinical trials have been conducted or are still recruiting volunteers to test many hypotheses of clinical effects implicating fucoxanthin, alone or combined with other compounds (Table 5). These studies are listed in the “ClinicalTrials.gov” database [131], a resource provided by the U.S. National Library of Medicine, containing privately and publicly funded clinical studies conducted around the world.

## 10. Discussion

The hallmarks of cancer, first described in 2000, comprise six biological capabilities acquired during the multistep development of human tumors: self-sufficiency in growth signals, insensitivity to growth inhibiting signals, the ability to avoid apoptosis and to replicate indefinitely, the induction of angiogenesis, and the ability to form metastases [132]. Later, two distinctive emerging capacities were added, i.e., the deregulation of cellular energy metabolism and the ability to avoid destruction by the immune system, without neglecting two characteristics favoring cancers, i.e., inflammation favoring tumors as well as instability and mutations of the genome [104].

On a structural level, metabolites of fucoxanthin are fucoxanthinol, which has been found to be more efficient than fucoxanthin, and amarouciaxanthin A [46]. Curiously, to our knowledge, the effects of amarouciaxanthin A on cancer prevention and therapy have not been clarified yet. However, considering those hallmarks, fucoxanthin and its derivatives appears to be a promising bioactive compound for treatment of cancer development and aggressiveness. Nonetheless, a few points need to be deepened, such as genomic instability through deregulation of DNA repair pathways, which can initiate cancer and finally result in resistance to chemo- and radio-therapy. It may be useful to study whether fucoxanthin has an inhibitory effect on DNA repair pathways, such as nucleotide excision repair, mismatch repair, and double-strand break repairs, which are involved in cancer therapy resistance [133,134].

Moreover, concerning angiogenesis, despite promising results showing that fucoxanthin downregulates the phosphorylation of FGF-2 and also involves decreased levels of VEGF-C, VEGF receptor-3, NF-κB, phospho-Akt, and phospho-PI3K in HLEC [88], further studies could elucidate the exact molecular mechanisms underlying the anti-angiogenic effect of fucoxanthin, especially those related to VEGF and FGF-2. This should lead to enhance the exploration of therapeutic potential of marine-derived molecules such as fucoxanthin. Indeed, angiogenesis is recognized as one of the hallmarks of cancer, a crucial step in the transition of tumors from a dormant stage to a malignant stage, and playing an essential role in tumor growth, invasion, and metastasis [104]. Key proteins implicated in the regulation of cellular adhesion, migration, and EMT are, for the most part, inhibited by fucoxanthin or its metabolite fucoxanthinol [20,99].

The role of fucoxanthin in the ability of tumor cells to deregulate cellular energy production, known as the “Warburg effect”, remains to be explored. It is described as the increased utilization of glycolysis rather than oxidative phosphorylation by cancer cells because of their energy requirement under normal oxygen conditions [135]. Zeaxanthin, another carotenoid, which is structurally similar to fucoxanthin, was shown to block hypoxia-induced VEGF secretion in cultured human retinal pigment epithelial cells and seems to modulate hypoxia-inducible factors-1 (HIF-1), which has been recognized as an important cancer drug target [136]. Extensive studies are necessary to complete potential inhibitory effects of fucoxanthin on the Warburg effect but also on two other hallmarks: enabling replicative immortality and the capacity of cancer cells to avoid immune destruction.

Fucoxanthin and more importantly its metabolite fucoxanthinol are able to reduce NF-κB activation [19,53,78,80,98], which favor apoptosis. NF-κB is also implicated in inflammation, which promotes cell proliferation and is linked to carcinogenesis. Indeed, inflammatory responses play decisive roles at different stages of tumor development [104]. Interestingly, Lin et al. found that *D. salina* carotenoids extract induced a downregulation of the expression of pro-inflammatory genes and a decrease of NF-κB activation in an antiviral context [123]. In addition, a few studies highlighted the anti-inflammatory potential of fucoxanthin in lipopolysaccharide (LPS)-induced inflammation [25,137]. In vivo, fucoxanthin was also found to decrease the expression of inflammatory cytokines in a prophylactic manner in the LPS-induced sepsis mouse model [42]. Furthermore, in combination with rosmarinic acid, fucoxanthin seems to exert anti-inflammatory effects by downregulating NRLP3-inflammasome and increasing the NFE2L2/Nrf2 signaling pathway [15]. However, further investigations are necessary to evaluate more precisely the anti-inflammatory effects of fucoxanthin in bacterial and especially viral infection, as well as its involvement in tumor progression.

The knowledge of the correlation between inflammation and tumor development should give the opportunity of developing new candidates for therapeutics. This could be used in combination with conventional therapeutic approaches, such as chemotherapy, radiotherapy, and targeted therapy [138]. Current findings suggest that fucoxanthin and its derived compounds could be effective for anticancer treatment and these carotenoids could be useful to support therapeutic strategies in combination with common cancer drugs. As described above (Section 5), fucoxanthin leads to improvement of the chemotherapeutic efficacy of cisplatin by targeting the DNA repair pathway [68]. Furthermore, fucoxanthin has been shown to act in synergy with other conventional drugs, such as troglitazone, a specific ligand for peroxisome proliferator-activated receptor (PPAR) gamma. Both drugs had a cytotoxic effect on Caco-2 cells while its separated administration had no effects [72]. In the same way, Ye et al. demonstrated that the use of a combined treatment with fucoxanthin and TRAIL could be a useful strategy against TRAIL-resistant cervical cancer by acting on the upregulation of the PI3K/Akt pathway [83,139]. Furthermore, Eid et al. also showed that fucoxanthin enhanced, synergistically, cytotoxicity induced by doxorubicin in human colorectal adenocarcinoma cells, by inhibiting the function of the ATP-Binding Cassette transporters [140]. These studies led to suggest that these biomolecules could be effective for cancer therapy as chemosensitizers, adjuvants, or even as active substances.

## 11. Conclusions

Cellular and animal studies have shown that fucoxanthin has anticancer effects. However, investigation of this role in humans is lacking. Clinical trials are required to assess the effect of fucoxanthin in close connection with the study of the mechanisms involved in the antitumoral action of fucoxanthin. Moreover, anticancer effects of fucoxanthin are regulated by several mechanisms leading to cell cycle arrest, induction of cell death and DNA damages, inhibition of metastasis-related migration, invasion and epithelial–mesenchymal transition, anti-angiogenic, and anti-inflammatory effects. Furthermore, combined treatments of fucoxanthin or fucoxanthinol with usual anticancer treatments can support conventional therapeutic strategies by reducing drug resistance. Indeed, as an anticancer molecule, fucoxanthinol appears to be a more effective bioactive compound than fucoxanthin. Therefore, the potential use of fucoxanthinol and other fucoxanthin derivatives (such as apo-9′-fucoxanthinone and apo-13-fucoxanthinone) as co-adjuvant agents in the treatment of cancer should be further investigated. However, further studies are necessary for precise and complete inhibitory effects of fucoxanthin and its derivatives, but regarding these hallmarks, fucoxanthin appears to be a promising compound for cancer therapy.

## Authors Contributions

All authors contributed equally to writing—review and editing, and have read and agreed to the published version of the manuscript. 

## Figures and Tables

**Table 1:** Source and concentration of fucoxanthin in different algal samples.

	Class	Species	Fucoxanthin Yield (mg/g)	References
Macroalgae	*Phaeophyceae*	*Alaria crassifolia*	0.04 ^a^	[18]
		*Cladosiphon okamuranus*	-	[19,20]
		*Cystoseira hakodatensis*	1.53 ^a^	[18]
		*Dictyota coriacea*	6.42 ^a^	[21]
		*Eisenia bicyclis*	0.26 ^b^–0.41 ^a^	[18,22]
		*Fucus evanescens C. agardh*	0.017 ^a^	[23]
		*Fucus vesiculus*	0.26–1.24	[24]
		*Ishige okamurae*	-	[25]
		*Kjellmaniella crassifolia*	0.197 ^a^	[18]
		*Laminaria japonica*	0.03 ^b^–0.19 ^b^	[26,27]
		*Myagropsis myagroides*	9.01 ^a^	[21]
		*Padina tetrastromatica*	0.18 ^b^	[28]
		*Petalonia binghamiae*	0.43 ^b^–0.58 ^b^	[29]
		*Saccharina japonica*	-	[30]
		*Sargassum fusiformis*	0.01 ^a^–0.02 ^b^	[26,27]
		*Sargassum binderi*	0.73 ^a^	[31]
		*Sargassum duplicatum*	1.01 ^a^	[31]
		*Sargassum hemiphyllum*	-	[16]
		*Sargassum horneri*	1.10 ^a^	[18]
		*Sargassum plagyophyllum*	0.71 ^a^	[31]
		*Sargassum polycystum*	0.31 ^a^	[32]
		*Sargassum siliquastrum*	0.75 ^a^	[33,34]
		*Sargassum siliquosum*	1.41 ^a^	[32]
		*Scytosiphon lomentaria*	0.24 ^b^–0.56 ^b^	[29]
		*Sphaerotrichia divaricata*	0.11 ^a^–1.48 ^a^	[35]
		*Turbinaria decurrens*	0.65 ^a^	[36]
		*Turbinaria turbinata*	0.59 ^a^	[31]
		*Undaria pinnatifida*	0.11 ^b^–1.09 ^a^	[26,27]
Microalgae	*Bacillariophyceae*	*Cyclotella* sp.	0.7 ^a^–2.3 ^a^	[37]
		*Nitzschia* sp.	4.92 ^a^–5.5 ^a^	[22,37]
		*Paralia longispina*	1.4 ^a^	[37]
		*Phaeodactylum tricornutum*	8.55 ^a^–24.2 ^a^	[22,37]
	*Coccolithophyceae*	*Prymnesium parvum*	7.91 ^a^	[38]
	*Chromulinaceae*	*Chromulina ochromonoides*	1.32 ^a^	[39]
	*Chrysophyceae*	*Ochromonas* sp.	0.41 ^a^	[39]
		*Ochromonas danica*	3.16 ^a^	[38]
	*Coscinodiscophyceae*	*Chaetoceros calcitrans*	5.25 ^a^	[40]
		*Ochromonas gracilis*	2.24 ^a^	[22]
		*Odontella aurita*	21.67 ^a^	[41]
	*Mediophyceae*	*Conticribra weissflogii* ND8	6 ^a^	[42]
	*Prymnesiophyceae*	*Isochrysis* sp.	17 ^a^	[43]
		*Isochrysis affinis galbana*	18.23 ^a^	[22]
		*Isochrysis galbana*	6.04 ^a^	[22]
	*Raphidophyceae*	*Olisthodiscus luteus*	0.08 ^a^	[39]
	*Synurophyceae*	*Synura petersenii*	0.02 ^a^	[39]
		*Mallomonas* sp. SBV13	26.6 ^a^	[37]
		*Poterioochromonas malhamensis*	0.6 ^a^	[39]
	*Zygnematophyceae*	*Cylindrotheca closterium*	5.23 ^a^	[44]

^a^ data obtained from dry biomass; ^b^ data obtained from fresh biomass.

**Table 2:** In vitro effects of fucoxanthin on cancer and non-cancer cells.

Cell Type and Origin			Cell Lines	Concentration (µM)	Effects	References
Cancer cells	Lung	Human	NSCLC-N6 A549	7.6–60.7	Apoptosis	[66]
			A549 H1299	12.5–25–50	Cell cycle arrest (G0/G1 mainly + S)	[67]
	Liver	Human	HepG2	1–10	Apoptosis	[68]
			SK-Hep-1	1–20	Cell cycle arrest (G0/G1) Apoptosis	[64]
			HepG2	3.8–5.5	Cell cycle arrest (G1)	[59]
			HepG2	25	Cell cycle arrest (G0/G1)	[62]
			HepG2	~20 µg/mL *	Apoptosis	[69]
	Gastric	Human	SGC-7901	12.5–25–50	Apoptosis/Autophagy	[70]
			MGC-803	50–75	Cell cycle arrest (G2/M) Apoptosis	[56]
	Colorectal	Human	DLD-1 cells	5	Inhibition of epithelial–mesenchymal transition (EMT)	[71]
			Caco-2	7.6	Apoptosis	[72]
			HCT116 HT29	10–50–100	DNA damage	[73]
			WiDr	25–50	Cell cycle arrest (G0/G1) Apoptosis	[57]
			Caco-2	25	Apoptosis DNA damage	[74]
	Bladder	Human	T24	5–10	Cell cycle arrest (G0/G1) Apoptosis	[54]
			EJ-1	20	Apoptosis	[75]
	Prostate	Human	DU145 LNCap	3.8–5.5	Cell cycle arrest (G1)	[58,59]
			PC-3 DU145 LNCap	20	Apoptosis	[76]
			PC-3	20	Apoptosis	[77]
	Breast	Human	MCF 7 MD-MB-231	10	Apoptosis	[78]
			MCF 7	20	Protect against DNA damage	[79]
			MCF 7 MD-MB-231	20–30–40	Apoptosis	[80]
			MCF 7	25	Apoptosis/DNA damage	[74]
	Cervix	Human	HeLa	0.5	Apoptosis	[81]
		Rat	C6	6	Protect against DNA damage	[82]
		Human	HeLa	10–20–40	Cell cycle arrest (G0/G1)	[65]
			SiHa	20	Apoptosis	[83]
	Neural	Human	GOTO	7.6–15.2	G0/G1 arrest	[61]
			U251	20	Apoptosis	[84]
			U251/U87	25–50	Apoptosis/Inhibition of migration and invasion	[85]
	Lymphoma	Human	Raji Daudi Ramos BJAB L428 KM-H2 HDLM-2 L540	2.5–5	Cell cycle arrest (G1; at lower concentration) Apoptosis (at higher concentration)	[19]
			HHV-8 infected BCBL-1 and TY-1	5–10	Cell cycle arrest (G1)	[53]
	Leukemia	Human	HL-60	12.5–25	Apoptosis/DNA Damage	[74]
			HL-60	10	Apoptosis	[86]
			K562 TK6	10	Antiproliferative	[51]
			MT-2/MT-4 HUT-102 ED-40515(-)	10	Cell cycle arrest (G1) Apoptosis	[52]
			HL-60	15	Apoptosis	[25]
	Melanoma	Mouse	B16-F10	30	Inhibition of invasion and migration Growth inhibition	[30]
			B16-F10	50–100–200	Cell cycle arrest (G0/G1) Apoptosis	[55]
	Sarcoma	Human	Saos 2	20	Apoptosis	[20]
Non-cancer cells	Umbilical vein endothelial cells	Human	HUVEC	1–5	Anti-angiogenic	[14]
	Keratinocytes		HaCaT	5 **	Anti-inflammatory	[15]
	Umbilical vein endothelial cells		HUVEC	2.5–5–10–25–50–100	Anti-angiogenic	[87]
	Lymphatic endothelial cells		HLEC	25–50–100	Anti-angiogenic	[88]

* this concentration corresponds to an extract enriched with fucoxanthin and not pure fucoxanthin (cf. [69]). ** in combination with rosmarinic acid.

**Table 3:** Effects of fucoxanthin and fucoxanthinol effects on in vivo cancer models (mice).

Context			Dose	Administration	Effects	References
Fucoxanthin	Lung	Engrafted with A549 cells	5–15–50 mg/kg	Oral	Necrosis	[67]
	Liver	Carcinogenesis model	0.001% in drinking water	Oral	Inhibition of carcinogenesis	[89]
	Duodenal	Carcinogenesis models	0.005% in drinking water	Oral	Inhibition of carcinogenesis	[90]
		Carcinogenesis models	0.005% in drinking water	Oral	Inhibition of carcinogenesis	[91]
	Colorectal	Carcinogenesis models	30 mg/kg	Injection (stomach)	Anoikis	[92]
		Carcinogenesis models	0.01% in drinking water	Oral	Inhibition of carcinogenesis	[93]
	Breast	Engrafted with MDA-MB-231 cells	100 and 500 µmol/L; 100 µL/mouse	Injection	Anti-angiogenic	[88]
	Cervix	Engrafted with HeLa cells	10 and 20 mg/kg	Oral	Growth inhibition	[81]
	Lymphoma	Engrafted with B16-F10	150 mg/kg	Oral	Growth inhibition	[53]
	Melanoma	Engrafted with B16-F10	0.1 mg/mouse	Intra-peritoneal injection	Anti-metastasis	[30]
		Carcinogenesis models	200 nM/painting	Topical application (skin painting)	Inhibition of carcinogenesis	[91]
	Sarcoma	Engrafted with S180 cells	50 and 100 mg/kg	Oral	Apoptosis	[94]
		Engrafted with LM8 cells	200 mg/kg	Oral	Growth inhibition	[20]
	Glioblastoma	Engrafted with U87 cells	200 mg/kg	Oral	Growth inhibition	[85]
Fucoxanthinol	Lymphoma	Engrafted with HUT-102 cells	200 mg/kg	Oral	Growth inhibition	[52]
	Sarcoma	Engrafted with LM8 cells	200 mg/kg	Oral	Growth inhibition	[20]

**Table 4:** In vitro effects of fucoxanthinol on cancer and non-cancer cells.

Cell Type and Origin			Cell Lines	Concentration (µM)	Effects	References
Cancer cells	Colorectal cancer	Human	DLD-1	1–5	Anoikis	[92]
			DLD-1	2.5	Anoikis Inhibition of EMT	[99]
			HCT116	5	Apoptosis	[98]
			Caco-2	12.5–25	Apoptosis DNA damage	[74]
			CRC HR29 HCT 116	50	Inhibition of EMT	[99]
	Breast cancer	Human	MCF-7	12.5–25	Apoptosis DNA damage	[74]
			MCF 7 MDA-MB-231	20–30–40	Apoptosis	[80]
	Lymphoma	Human	Raji Daudi Ramos, BJAB L428 KM-H2 HDLM-2 L540	1.25–2.5	Cell cycle arrest (G1; at lower concentration) Apoptosis (at higher concentration)	[19]
			HHV-8 infected BCBL-1 TY-1	2.5–5	Cell cycle arrest (G1)	[53]
			MT-2 MT-4 HUT-102 ED-40515(-)	5	Cell cycle arrest (G1) Apoptosis	[52]
	Leukemia	Human	HL-60	6.25–12.5	Apoptosis Antiproliferative DNA Damage	[74]
	Sarcoma	Human	Saos 2	0.63–1.25	Inhibition of migration	[20]
				0.05–0.1	Inhibition of invasion	[20]
Non-cancer cells	-	Rat	Aortic ring	2.5–5–10–25	Anti-angiogenic	[87]

**Table 5:** Clinical trials involving Fucoxanthin.

NCT Number	Title	Conditions	Interventions	Phases	Study Designs	Start Date	Locations
NCT02875392	Fucoidan Improves the Metabolic Profiles of Patients With Non-alcoholic Fatty Liver Disease (NAFLD)	Non-alcoholic Fatty Liver Disease	Other: 275 mg Oligo Fucoidan +275 mg HS Fucoxanthin|Other: placebo pills	NA (unknown status)	Allocation: Randomized Intervention Model: Parallel Assignment Masking: Double (Participant, Care Provider) Primary Purpose: Treatment	November 2016	WanFangH, Taipei, Taiwan
NCT03625284	Oral Dietary Fucoxanthin-Rich Supplement for Liver Health	Non-Alcoholic Fatty Liver	Dietary Supplement: Placebo Dietary Supplement: FucoVital	NA (unknown status)	Allocation: Randomized Intervention Model: Parallel Assignment Masking: Quadruple (Participant, Care Provider, Investigator, Outcomes Assessor) Primary Purpose: Prevention	10 September 2018	Assaf-Harofeh Medical Center, Israël
NCT03613740	Effect of Fucoxanthin on the Components of the Metabolic Syndrome, Insulin Sensitivity and Insulin Secretion	Metabolic Syndrome	Drug: Fucoxanthin|Drug: Placebo	Phase 2 (still recruiting)	Allocation: Randomized Intervention Model: Parallel Assignment Masking: Double (Participant, Investigator) Primary Purpose: Treatment	30 September 2019	Instituto de Terapéutica Experimental y Clínica. Centro Universitario de Ciencias de la Salud. Guadalajara, Mexico
NCT04288544	Health Promoting Effects of the Microalgae Phaeodactylum Tricornutum	Human Nutrition, Omega-3 Fatty Acids, Microalgae Micronutrients	Dietary Supplement: Microalgae Dietary Supplement: Omega-3-fatty acid capsule Dietary Supplement: sea fish	NA (Enrolling by invitation)	Allocation: Non-Randomized Intervention Model: Crossover Assignment Masking: None (Open Label) Primary Purpose: Supportive Care	25 February 2020	lena Stiefvatter, Stuttgart, Germany University of Hohenheim, Stuttgart, Germany

NA: Not Applicable; NCT: Number Clinical Trials (identifier); Source “ClinicalTrials.gov” database [131].

## Abbreviations

AP-1	activator protein-1
ASC	apoptosis-associated speck-like protein
ATM	ataxia telangiectasia mutated protein
ATR	ataxia telangiectasia and Rad3-related protein
BMI	body mass index
CAT	catalase
CDK	cyclin-dependent kinase
CXCR4	C-X-C motif chemokine receptor 4
EGFR	epidermal growth factor receptor
EMT	epithelial–mesenchymal transition
ERCC1	excision repair cross complementation 1
ERK	extracellular signal-regulated kinase
FGF	fibroblast growth factor
HIF-1	hypoxia-inducible factor-1
HLEC	human lymphatic endothelial cells
HTLV-1	human T-cell leukemia virus type 1
HUVEC	human umbilical vein endothelial cells
IL	Interleukin
JNK	Jun-N-terminal kinase
LC3	microtubule-associated protein 1A/1B-light chain 3
LPS	lipopolysaccharide
MAPK	mitogen activated kinase
MCP-1	monocyte chemoattractant protein-1
MMP	matrix metalloproteinase
mTOR	mechanistic target of rapamycin
NAC	N-acetyl cysteine
NER	nucleotide excision repair
NFE2L2/Nrf2	nuclear factor (erythroid-derived) like-2
NF-κB	nuclear factor κB
NLRP3	NOD-like receptor family, pyrin domain containing 3
PARP	poly-ADP-ribose polymerase
PI3K	phosphoinositide-3 kinase
p-FAK	phosphorylated focal adhesion kinase
PGI2	prostacyclin
pRb	retinoblastoma protein
PPAR	peroxysome proliferator-activated receptor
PTEN	phosphatase and tensin homolog protein
PUMA	p53 upregulated modulator of apoptosis
ROS	reactive oxygen species
SOD	superoxide dismutase
STAT	signal transducer and activator of transcription 3
TNFα	tumor necrosis factor α
TRAIL	tumor necrosis factor-related apoptosis-inducing ligand
UCP1	uncoupling protein-1
VEGF	vascular endothelial growth factor
XIAP	X-linked inhibitor of apoptosis
